# Gene model for the ortholog of *Thor* in *Drosophila ananassae*

**DOI:** 10.17912/micropub.biology.000854

**Published:** 2026-07-06

**Authors:** Madeline L. Gruys, Jhilam Dasgupta, Joshua Williams, Emile Moura Coelho da Silva, Jacqueline Wittke-Thompson, Chinmay P. Rele, Laura K. Reed

**Affiliations:** 1 The University of Alabama, Tuscaloosa, AL USA; 2 University of St. Francis, Joliet, IL USA; 3 University of Evansville, Evansville, IN USA

## Abstract

Here is presented a gene model for the ortholog of Thor ( *Thor* ) in the *D. ananassae* May 2011 (Agencourt dana_caf1/DanaCAF1) Genome Assembly (GenBank Accession: GCA_000005115.1 ) of *Drosophila ananassae* . This ortholog was characterized as part of a developing dataset to study the evolution of the Insulin/insulin-like growth factor signaling pathway (IIS) across the genus *Drosophila* using the Genomics Education Partnership gene annotation protocol for Course-based Undergraduate Research Experiences.

**Figure 1. Genomic neighborhood and gene model for Thor in Drosophila ananassae:** **
(A) Synteny comparison of the genomic neighborhoods for *Thor* in *Drosophila melanogaster* and *D.* *ananassae* .** Thin underlying arrows indicate the DNA strand within which the target gene– *Thor* –is located in *D. melanogaster* (top) and *D. ananassae* (bottom). Thin arrows pointing to the right indicate that *Thor* is on the positive (+) strand in both *D. ananassae* and *D. melanogaster* . The wide gene arrows pointing in the same direction as *Thor* are on the same strand relative to the thin underlying arrows, while wide gene arrows pointing in the opposite direction of *Thor* are on the opposite strand relative to the thin underlying arrows. White gene arrows in *D. ananassae* indicate orthology to the corresponding gene in *D. melanogaster* . Gene symbols given in the *D. ananassae* gene arrows indicate the orthologous gene in *D. melanogaster* , while the locus identifiers are specific to *D. ananassae* . **(B) Gene Model in GEP UCSC Track Data Hub (Raney et al. 2014).** The coding-regions of *Thor* in *D. ananassae* are displayed in the User Supplied Track (black); coding CDSs are depicted by thick rectangles and introns by thin lines with arrows indicating the direction of transcription. Subsequent evidence tracks include BLAT Alignments of NCBI RefSeq Genes (dark blue, alignment of Ref-Seq genes for *D. ananassae* ), Spaln of *D. melanogaster* Proteins (purple, alignment of Ref-Seq proteins from *D. melanogaster* ), Transcripts and Coding Regions Predicted by TransDecoder (dark green), RNA-Seq from Adult Females and Adult Males (red and light blue, respectively; alignment of Illumina RNA-Seq reads from *D. ananassae* ), and Splice Junctions Predicted by regtools using *D. ananassae* RNA-Seq (Graveley *et al.* , 2011; SRP006203 , SRP007906 ; PRJNA257286 , PRJNA388952 ). The splice junction shown in red (JUNC00011951) has a read-depth of 4677. **(C) Dot Plot of Thor-PA in *D. melanogaster* ( *x* -axis) vs. the orthologous peptide in *D. ananassae* ( *y* -axis).** Amino acid number is indicated along the left and bottom; CDSs number is indicated along the top and right, and CDSs are also highlighted with alternating colors. The black box X highlights a repeating amino acid sequence in that region. **(D) Protein Alignment Thor-PA in *D. melanogaster* and the orthologous peptide in *D. ananassae* .** The alternating colored rectangles represent adjacent CDSs. The symbols in the match line denote the level of similarity between the aligned residues. An asterisk (*) indicates that the aligned residues are identical. A colon (:) indicates the aligned residues have highly similar chemical properties—roughly equivalent to scoring > 0.5 in the Gonnet PAM 250 matrix (Gonnet *et al.* , 1992). A period (.) indicates that the aligned residues have weakly similar chemically properties—roughly equivalent to scoring > 0 and ≤ 0.5 in the Gonnet PAM 250 matrix. A space indicates a gap or mismatch when the aligned residues have a complete lack of similarity—roughly equivalent to scoring ≤ 0 in the Gonnet PAM 250 matrix. The amino acid sequence shows there is a small repeat at the end of CDS one (TPGGT) as shown in the 2 boxes, corresponding to Box X in the dot plot. **(E) End of first CDS of *Thor* in *D. melanogaster* displaying conservation of TPGGT repeats.** Evidence tracks include base positions shown in black at the top of the browser, amino acid sequence (grey blocks), FlyBase Protein-Coding Genes (blue), ROAST Alignment and Conservation (36 RefSeq *Drosophila* Genomes) (burgundy), Basewise Conservation of 36 *Drosophila* Genomes generated by phastCons and phyloP shown in green and dark blue, respectively; ROAST Alignments of 36 *Drosophila* species with species listed on the left in dark blue and amino acid sequence in pale blue. Boxes in black denoted X1 and X2i highlight the conservation of the amino acid repeat (TPGGT) at the end of the first CDS of *Thor* across 36 *Drosophila* species. The yellow highlighted box labeled Y on the left encloses the sequence in *D. ananassae* , with X2i encompassing only a portion of the TPGGT repeat, (TPGG). **(F) Beginning of second CDS of *Thor* in *D. melanogaster* displaying conservation of amino acid sequence.** Evidence tracks are identical to those in Figure 1E. The box in black denoted X2ii highlights the conservation of the amino acid repeat (TPGGT) at the beginning of the second CDS of *Thor* in *D. melanogaster* , featuring the remaining amino acids of the repeat (T). The continuation of the yellow highlighted box Y encloses the sequence in *D. ananassae* .

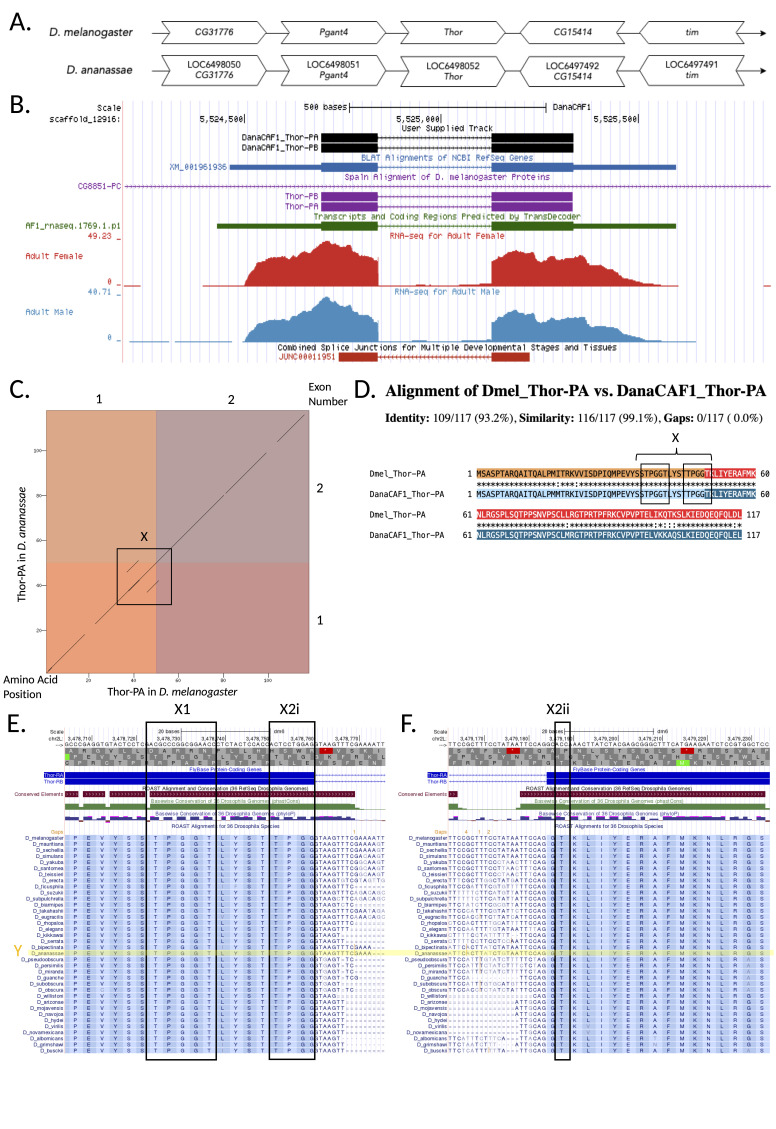



## Description

**Table:** 

*This article reports a predicted gene model generated by undergraduate work using a structured gene model annotation protocol defined by the Genomics Education Partnership (GEP; thegep.org) for Course-based Undergraduate Research Experience (CURE). The following information may be repeated in other articles submitted by participants using the same GEP CURE protocol for annotating Drosophila species orthologs of Drosophila melanogaster genes in the insulin signaling pathway.* "In this GEP CURE protocol students use web-based tools to manually annotate genes in non-model *Drosophila* species based on orthology to genes in the well-annotated model organism fruitfly *Drosophila melanogaster* . The GEP uses web-based tools to allow undergraduates to participate in course-based research by generating manual annotations of genes in non-model species (Rele et al., 2023). Computational-based gene predictions in any organism are often improved by careful manual annotation and curation, allowing for more accurate analyses of gene and genome evolution (Mudge and Harrow 2016; Tello-Ruiz et al., 2019). These models of orthologous genes across species, such as the one presented here, then provide a reliable basis for further evolutionary genomic analyses when made available to the scientific community.” (Myers et al., 2024). “The particular gene ortholog described here was characterized as part of a developing dataset to study the evolution of the Insulin/insulin-like growth factor signaling pathway (IIS) across the genus *Drosophila* . The Insulin/insulin-like growth factor signaling pathway (IIS) is a highly conserved signaling pathway in animals and is central to mediating organismal responses to nutrients (Hietakangas and Cohen 2009; Grewal 2009).” (Myers et al., 2024). “ *D* . *ananassae* (NCBI:txid7217) is part of the *melanogaster* species group within the subgenus *Sophophora* of the genus *Drosophila* (Sturtevant 1939; Bock and Wheeler 1972). It was first described by Doleschall (1858). *D. ananassae* is circumtropical (Markow and O'Grady 2005; https://www.taxodros.uzh.ch, accessed 1 Feb 2023), and often associated with human settlement (Singh 2010). It has been extensively studied as a model for its cytogenetic and genetic characteristics, and in experimental evolution (Kikkawa 1938; Singh and Yadav 2015).” (Lawson et al., 2024).

We propose a gene model for the *D. ananassae* ortholog of the *D. melanogaster* Thor *( Thor )* gene. The genomic region of the ortholog corresponds to the uncharacterized protein LOC6498052 (RefSeq accession XP_001961972.1 ) in the dana_caf1 Genome Assembly of *D. ananassae* (GenBank Accession: GCA_000005115.1 , Drosophila 12 Genomes Consortium et al., 2007). This model is based on RNA-Seq data from *D. ananassae* (Graveley et al., 2011; SRP006203 , SRP007906 ; PRJNA257286 , PRJNA388952 ) and *Thor* in *D. melanogaster* using FlyBase release FB2022_04, ( GCA_000001215.4 ; Larkin et al., 2021; Gramates et al., 2022; Jenkins et al., 2022).

*Thor* ( *Thor* ; also known as *4E-BP* ), a core component of the insulin signaling pathway, encodes a eukaryotic translation initiation factor 4E binding protein that is controlled by the product of *mTor* (Bernal and Kimbrell 2000; Marr II et al., 2007). The *Drosophila* forkhead transcription factor ( *dFOXO* ) activates *Thor* transcription and contributes to translation regulation, response to environmental stress, and cell growth regulation (Tettweiler et al., 2005; Miron et al., 2001). *Thor* is an effector of *PI(3)K* / *Akt* signaling and cell growth in *Drosophila* (Miron et al., 2001) and participates in host immune defense by connecting a translational regulator with innate immunity (Bernal and Kimbrell 2000).

***Synteny***

The reference gene, *Thor ,* is found on chromosome 2L in *D. melanogaster* and is flanked upstream by *CG31776* and Polypeptide N-Acetylgalactosaminyltransferase 4 *(Pgant4)* and downstream by *CG15414* and timeless *(tim)* . The *tblastn* search of *D. melanogaster* Thor-PA (query) against the *D. ananassae* (GenBank Accession: GCA_000005115.1 ) Genome Assembly (database) placed the putative ortholog of *Thor* within scaffold_12916 ( CH902620.1 ) at locus LOC6498052 ( XP_001961972.1 )— with an E-value of 1e-78 and a percent identity of 93.16%. Furthermore, the putative ortholog is flanked upstream by LOC6498050 ( XP_001961970.1 ) and LOC6498051 (XP_ 001961971.1 ), which correspond to *CG31776* and *Pgant4* in *D. melanogaster* (E-value: 0.0 and 0.0; identity: 47.45% and 74.40%, respectively, as determined by *blastp* ; Figure 1A, Altschul et al., 1990). The putative ortholog of *Thor* is flanked downstream by LOC6497492 ( XP_044572519.1 ) and LOC6497491 ( XP_032307461.1 ), which correspond to *CG15414* and *tim* in *D. melanogaster* (E-value: 2e-108 and 0.0; identity: 83.33% and 85.86%, respectively, as determined by *blastp* ). The putative ortholog assignment for *Thor* in *D. ananassae* is supported by the following evidence: The genes surrounding the *Thor* ortholog are orthologous to the genes at the same locus in *D. melanogaster* and local synteny is completely conserved, verified by e-values and percent identities, so we conclude that LOC6498052 is the correct ortholog of *Thor* in *D. ananassae* ( Figure 1A ).

***Protein Model***

*Thor* in *D. ananassae* has two mRNA transcripts (Thor-RA, Thor-RB), which encode identical protein-coding isoforms (Thor-PA; Thor-PB; Figure 1B ). These isoforms (Thor-PA, Thor-PB) contain two CDSs. Relative to the ortholog in *D. melanogaster* , the CDS number is conserved. The sequence of Thor-PA in *D. ananassae* has 93.16% identity (E-value: 1e-78) with the protein-coding isoform Thor-PA in *D. melanogaster* , as determined by *blastp* ( Figure 1C ). Consistent with the *blastp* search result which shows 93.16% identity between *D. melanogaster* Thor-PA and the *D. ananassae* protein model as well as the low sensitivity parameters used to generate the dot plot (i.e., word size = 3; neighborhood threshold = 11), the dot plot of the two protein sequences contain multiple small gaps along the diagonal ( Figure 1C ). The dots on either side of the diagonal at the end of CDS one in the dot pot (Box X, Figure 1C ) indicate the presence of two identical copies of the sequence TPGGT, as shown in the two boxes labeled B in the protein alignment (Box X, Figure 1D ). Coordinates of this curated gene model are stored by NCBI at GenBank/BankIt (accession BK064668 , BK064669 ). These data are also archived in the CaltechDATA repository (see “Extended Data” section below).

***Special characteristics of the protein model***

**TPGGT Repeat:** The dots on either side of the diagonal at the end of CDS one in the dot pot ( Figure 1C ) indicates the presence of two identical copies of the sequence TPGGT, as shown in the two boxes labeled X1 and X2 in the protein alignment ( Figure 1D ). This small repeat is conserved in many *Drosophila* species as shown by the black boxes denoted X1, X2i and X2ii, with *D. ananassae* highlighted in the yellow box, Y ( Figure 1E and Figure 1F ).

## Methods

Detailed methods including algorithms, database versions, and citations for the complete annotation process can be found in Rele et al. (2023). Briefly, students use the GEP instance of the UCSC Genome Browser v.435 ( https://gander.wustl.edu ; Kent WJ et al., 2002; Navarro Gonzalez et al., 2021) to examine the genomic neighborhood of their reference IIS gene in the *D. melanogaster* genome assembly (Aug. 2014; BDGP Release 6 + ISO1 MT/dm6). Students then retrieve the protein sequence for the *D. melanogaster* reference gene for a given isoform and run it using *tblastn* against their target *Drosophila* species genome assembly on the NCBI BLAST server ( https://blast.ncbi.nlm.nih.gov/Blast.cgi ; Altschul et al., 1990) to identify potential orthologs. To validate the potential ortholog, students compare the local genomic neighborhood of their potential ortholog with the genomic neighborhood of their reference gene in *D. melanogaster* . This local synteny analysis includes at minimum the two upstream and downstream genes relative to their putative ortholog. They also explore other sets of genomic evidence using multiple alignment tracks in the Genome Browser, including BLAT alignments of RefSeq Genes, Spaln alignment of *D. melanogaster* proteins, multiple gene prediction tracks (e.g., GeMoMa, Geneid, Augustus), and modENCODE RNA-Seq from the target species. Detailed explanation of how these lines of genomic evidenced are leveraged by students in gene model development are described in Rele et al. (2023). Genomic structure information (e.g., CDSs, intron-exon number and boundaries, number of isoforms) for the *D. melanogaster* reference gene is retrieved through the Gene Record Finder ( https://gander.wustl.edu/~wilson/dmelgenerecord/index.html ; Rele et al *.,* 2023). Approximate splice sites within the target gene are determined using *tblastn* using the CDSs from the *D. melanogaste* r reference gene. Coordinates of CDSs are then refined by examining aligned modENCODE RNA-Seq data, and by applying paradigms of molecular biology such as identifying canonical splice site sequences and ensuring the maintenance of an open reading frame across hypothesized splice sites. Students then confirm the biological validity of their target gene model using the Gene Model Checker ( https://gander.wustl.edu/~wilson/genechecker/index.html ; Rele et al., 2023), which compares the structure and translated sequence from their hypothesized target gene model against the *D. melanogaster* reference gene model. At least two independent models for a gene are generated by students under mentorship of their faculty course instructors. Those models are then reconciled by a third independent researcher mentored by the project leaders to produce the final model. Note: comparison of 5' and 3' UTR sequence information is not included in this GEP CURE protocol (Gruys et al., 2025).

## Data Availability

Description: GFF, FASTA, PEP. Resource Type: Model. DOI: https://doi.org/10.22002/7z36m-eek13
